# Varicocele and Its Radical Cure

**Published:** 1894-01

**Authors:** Samuel E. Milliken

**Affiliations:** Lecturer on Surgery at the New York Polyclinic; New York


For Texas Medical Journal.

### VARICOCELE AND ITS RADICAL CURE.

BY SAMUEL E. MILLIKEN, M. D.,
Lecturer on Surgery at the New York Polyclinic.

[Read at Austin District Medical Society, December 21, 1893.]

IN CHOOSING a subject to present to your Society, I believe that none can be found, within the domain of my own work, of more interest than the radical cure of varicocele. While it is a condition which has never, to my knowledge, caused death, all who have seen the reflex phenomena occasioned by the presence of varicocele, in its various stages, must admit that means of relief should be instituted, and if palliative measures fail, operation should be performed. Since the writer has little confidence in the former where the varicose condition of the veins has progressed with the consequent atrophy of the testis, this paper will be devoted to its radical cure.

With strict asepsis, the operation is not only safe, but relief can be assured the patient in every instance.

### OPERATION.

After having shaven the pubis, scrotum and perineum, the genitals are thoroughly cleansed with soap and water, then with ether, and lastly with bichloride of mercury (1-3000). Under ether anæsthesia, an incision one and a half or two inches long is made along the cord structures, exposing them after the skin is cut, treat by dividing the respective layers on a grooved director, so as not to cut a vein. By taking that precaution, the whole mass of vessels can be brought well into view, and all that remains is to isolate the vas deferens and its immediate blood vessels and nerve supply, while the varicose mass of veins are separated for a distance of one or two inches, tied at each extremity with strong catgut, and the intervening segment excised. The respective ligatures are tied together so as to approximate the freshly cut ends, and thus relieve the vas and its accompanying structures of the weight of the testis. The wound is closed with catgut, and no drain is used.

*Excision* of the lower end of the scrotum is unnecessary, for when once the diseased vessels have been removed, the scrotum will soon be found retracted to its normal length.

*Subcutaneous* ligation of the veins, while yet practiced by a few, is rapidly falling into disuse, as are many such procedures of ten years ago. It is needless to say that such blind surgery is risky, but however skillfully performed, the large inflammatory mass remains for months. The safety of the cord cannot be assured, unless it be constantly in view of the operator.

CASES.

Case 1. Edward R., 22 years of age, expressman, left varicocele of five (5) years standing, operated upon June 30, 1890. Excision of veins and silk ligatures used. Small drain inserted.

July 2. Drain removed and wound dressed.

July 5. Small sinus remains at site of drain. Patient left the bed wearing suspensory bandage.

July 18. Wound healed.

July 31. Discharge began from former sinus, but very slight.

August 29. Entirely healed, only small enlargement of testis remains.

Case 2. John B., 33 years of age, machinist, left varicocele of years duration. Operated upon July 16, 1890. Silk ligatures again employed, also small rubber drain inserted. Catgut for skin wound.

July 17. Drain removed.

July 20. Allowed to get out of bed; union except at point of drain.

September 1. Sinus healed.

In the above two cases it can clearly be seen that the sinuses were the result of drainage tubes and silk for ligating the veins.

Case 3. John B., Jr., 18 years old. Life insurance clerk. Varicocele of three years standing.

Dec. 28, 1891. Operation—catgut employed throughout; small piece of gauze for drain. Drain removed on the second day.

January 14, 1892. All dressings left off. Suspensory bandage to be worn.

August 1, 1892. Each side found symmetrical.

Case 4. Herman H., 22 years. Same operation as case 3, on February 9, 1891. Discharged from hospital at the end of two weeks.

In five other cases operated upon since, where catgut was employed throughout, and without drainage tube, all the wounds united by first intention, and no sinuses followed.

One out of ten cases, a physician, infected the wound, which caused it three weeks delay in healing, as it had to unite by granulation.

CONCLUSIONS.

1st. Complete excision of the diseased veins is preferable to subcutaneous ligation.

2nd. Absorbable sutures and ligatures should only be employed.

3rd. Strict asepsis, and no drain will be found necessary.

4th. The patient can with safety leave the bed in one week.

